# Novel and Automatic Rice Thickness Extraction Based on Photogrammetry Using Rice Edge Features

**DOI:** 10.3390/s19245561

**Published:** 2019-12-16

**Authors:** Yuchen Kong, Shenghui Fang, Xianting Wu, Yan Gong, Renshan Zhu, Jian Liu, Yi Peng

**Affiliations:** 1 School of Remote Sensing and Information Engineering, Wuhan University, Wuhan 430079, China; yuchenk0219@whu.edu.cn (Y.K.); gongyan@whu.edu.cn (Y.G.); liu_jian@whu.edu.cn (J.L.); ypeng@whu.edu.cn (Y.P.); 2 Lab for Remote Sensing of Crop Phenotyping (LRSCP), Wuhan University, Wuhan 430079, China; renshan8@whu.edu.cn; 3 College of Life Sciences, Wuhan University, Wuhan 430079, China

**Keywords:** rice grain, thickness, crop phenotyping, digital image processing, photogrammetry

## Abstract

The dimensions of phenotyping parameters such as the thickness of rice play an important role in rice quality assessment and phenotyping research. The objective of this study was to propose an automatic method for extracting rice thickness. This method was based on the principle of binocular stereovision but avoiding the problem that it was difficult to directly match the corresponding points for 3D reconstruction due to the lack of texture of rice. Firstly, the shape features of edge, instead of texture, was used to match the corresponding points of the rice edge. Secondly, the height of the rice edge was obtained by way of space intersection. Finally, the thickness of rice was extracted based on the assumption that the average height of the edges of multiple rice is half of the thickness of rice. According to the results of the experiments on six kinds of rice or grain, errors of thickness extraction were no more than the upper limit of 0.1 mm specified in the national industry standard. The results proved that edge features could be used to extract rice thickness and validated the effectiveness of the thickness extraction algorithm we proposed, which provided technical support for the extraction of phenotyping parameters for crop researchers.

## 1. Introduction

Rice is one of the most important food crops for humans [1]. According to statistics, about half of the people in the world depend on rice as their staple food [2]. A significant part of rice quality evaluation is the analysis of rice dimension parameters [3,4,5], such as length, width and thickness of rice kernel. The length and width of rice determine the shape traits of rice [4,6] while the thickness reflects the fullness of rice and affects the rice fissuring [7]. In addition, researchers have made a lot of progress in determining the genes that determine the length or width of rice [8], but in terms of thickness, there is little. The main reason is that there is no fast and efficient way to measure the thickness of rice. Therefore, scholars have become increasingly interested in crop phenotypes [9,10] and phenotyping parameters such as rice thickness, the measurement of which plays an important role in the study of rice growth and reproduction, genetic breeding [11,12] and so on.

At present, the traditional extraction of rice dimensions parameters depends on manual measurement with tools such as the micrometer screw gauge or vernier calipers [5,13]. Although this method has high precision, the measurement results are not only subjective, but also require a lot of time and manpower, which is inefficient and difficult. Usually agricultural researchers only extract the length and width of rice, while there is very little research dealing with measurement of rice thickness.

In order to automatically acquire 3D information of rice such as the length, width and thickness at the same time, scholars have studied the laser point cloud [14]. Li et al. [15] used a 3D laser scanner to obtain point cloud data of the surface of rice, and constructed an external rectangular parallelepiped of the rice according to the oriented bounding box (OBB) algorithm in order to obtain the length, width and thickness. This method still relies on manual operation in the acquisition of point cloud data, and the work of data preprocessing takes about two hours, which is too time consuming. Huang et al. [16] also used a point cloud to automatically extract the phenotypic data of grains. The axis-aligned bounding box (AABB) algorithm was adopted to extract the length and width of rice. The precision is very high, but it is necessary to purchase a specific laser-scanning instrument, which is expensive and not conducive to promotion.

With continuous development of image-processing technology in the food and agricultural industry [17,18,19,20], scholars began to study how to more easily and automatically extract the dimensions of the phenotyping parameters of rice [21]. Ling [22] believed that the point on the rice edge, which is farthest from the centroid of rice, is one of the end points of the long axis, and the nearest point is one of the end points of the short axis. Extend the line-segments connecting the end point and the centroid respectively, and they will intersect with the edge to form a long and a short, two line-segments, the length of which is the length and width of rice. Ali S F et al. [23] created a circumscribed rectangle for the rice, with the length of the diagonal of the rectangle as the length of rice, and the sum of the two shortest distances from the edge to the centroid as the width of rice. Huang [24] regarded the two farthest points on the edge as the two end points of the long axis, the length of which is the length of rice. The length of the projection of the rice in the normal direction of the long axis is the width of rice. Wan [25] and Ma [26] used the length and width of the smallest circumscribed rectangle of rice as the length and width of rice. Zhang [27] used the extreme length of rice projected in a certain direction during rotation as the length and width of rice. So far, these methods based on image processing or machine vision [28] have been relatively mature, but they are limited to the 2D level, only extracting the length and width of rice. Only a few scholars put grains into a V-groove and obtain the thickness from side images [29]. This method needs to put the samples in a row manually, and each time the amount of grain samples allowed is small and limited. The length and width information cannot be extracted simultaneously. Essentially, it is still a 2D image-processing problem.

According to the principle of stereo vision [30,31], it is necessary to match the corresponding points from two images in order to obtain the 3D thickness information from 2D images [32]. But due to the fact that the shape of rice is similar to each other and the surface texture is scarce, it is impossible to realize matching directly using the traditional matching algorithm [33], which greatly increases the difficulty of extracting rice thickness from images.

Based on the above analysis, a method for automatic extraction of rice thickness is proposed in this paper. According to the hypothesis that the average height of the edges of multiple rice is half of the thickness of rice, the target is transformed from the thickness to the height of the rice edge. Then, aiming at the problem that the edges of rice still have no obvious texture features, a matching method based on edge shape is developed, in which the shape features is used instead of the texture to realize the matching. The objectives of this paper were to: (i) explore and demonstrate the concept of using edge features to extract rice thickness automatically, and (ii) validate the method using different kinds of rice grain with different samples and different base-height ratios. It turned out that this method is reliable for obtaining rice thickness and showed great potential to contribute to phenotyping researches.

## 2. Materials and Methods 

### 2.1. Experimental Samples

A total of 160 samples from six kinds of rice or grain were used in the experiment (Table 1). The rice morphology of different varieties is quite different from each other so that the experimental result is universal and flexible. Since some rice seeds do not need to be hulled, experiments were carried out as well to find out whether the method in this paper is applicable to grains.

For each variety of rice, 20 samples in good shape were randomly selected. The long distance between the ends is the length of rice. The way to distinguish width and thickness is: to place rice naturally on a plane, the distance perpendicular to the plane is called the thickness, and the distance between the back and the belly of rice and parallel to the plane is the width. The thickness of each rice was measured one by one with a vernier caliper with a precision of 0.02 mm. The average value was taken as the true value of rice.

### 2.2. Experimental Equipment

**Figure 1:**
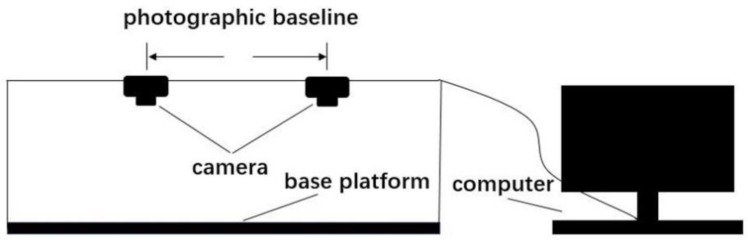
Experiment equipment.

**Figure 2:**
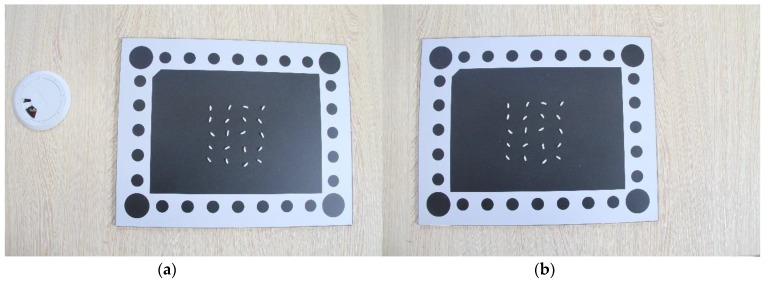
Experimental equipment: (**a**) left image of the stereo pairs; (**b**) right image of the stereo pairs.

The image acquisition system is shown in Figure 1. Experiments were conducted in an adequate-and-uniform-light environment. The rice was randomly seeded on the background board on the base platform, which belongs to the overlapping field of view of the two cameras (Canon EOS 7D) above. Two cameras were about 35 centimeters above the rice and captured rice images at the same time, the resolution of which was 5184 × 3456. The two images (for example, Figure 2) were transmitted to the computer in real time. MATLAB programming was used for subsequent processing and analysis. 

Compared with the image taken above the rice, the rice edges on the two images may not completely correspond when the rice is taken from different positions due to the problem of occlusion, which makes the edge matching experience deviations. The photographic baseline is the distance between two cameras when capturing the images. If the baseline is too long, the edge of rice may be blocked by the highest point, resulting in ‘dead ends’, while the accuracy of intersection angle will be lost if the baseline is too short. 

Therefore, the two cameras are not fixed all the time during the measurement experiment because different lengths of photographic baselines were selected to study the effect of the distance between two cameras on the accuracy of thickness. 

It is worth mentioning that the experimental results are all based on an appropriate base-height ratio to mitigate the effect as much as possible. The tests we performed and the discussion on the base-height ratio will be presented in detail in Section 3.5.

### 2.3. Camera Calibration

Since this study involves dimensions computation, the elements of interior orientation and distortion coefficients of the camera are needed. The elements of interior orientation refer to the parameters of the camera lens center relative to the image position [34], including the position $x_{0}$ and $y_{0}$ of the principal point of photograph (the foot point of the optical axis on the imaging plane) relative to the center of the image and the vertical distance *f* from the lens center to the imaging plane (also called the principal distance) [35]. Since the elements of interior orientation and distortion coefficients are inherent parameters of the camera, they will not change during the follow-up multiple uses, which can be used directly, if a check is made before shooting rice. The way to obtain the elements of interior orientation and distortion coefficients in this paper is the camera calibration method with the single-plane checkerboard proposed by Zhang in 1998 [36]: taking the black-and-white chessboard diagram as the calibration board, 10–20 pictures of the board were taken with the camera at different positions, postures and angles to solve the camera’s elements of interior orientation and distortion coefficients.

### 2.4. Basic Hypothesis of Edge Height

**Figure 3:**
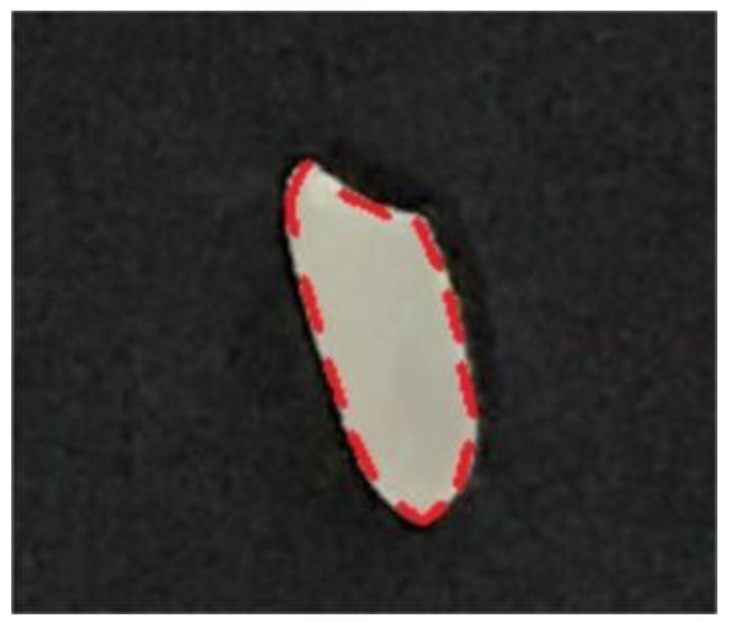
Top view of rice.

**Figure 4:**
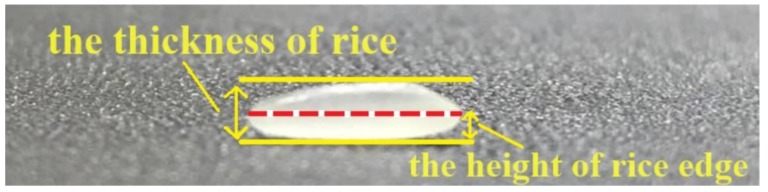
Side view of rice.

Observing the top view of rice (Figure 3), we know the red dotted line is its outline edge. Combined with the side view (Figure 4), you can see that the edge is about half the height of the whole rice. Suppose a certain rice has a thickness of *H*, and it has the difference of ‘front and back side’. The height from the edge to the ground is *h* with the front side facing upward, and the height from the edge to the ground is *H*-*h* with the back side facing upward. Since the shape of rice is generally symmetrical, when multiple rice falls randomly on the ground, the probability of the occurrence of either side facing upward is 1/2, and the expectation of the height from the edge to the ground is:

(1) $$\frac{1}{2}h+\frac{1}{2}\left(H-h\right)=\frac{1}{2}H$$

This means that when more than one rice is sprinkled randomly, the average height of the edges of rice is half of the thickness of rice. According to this assumption, the thickness of rice can be obtained indirectly by calculating its height of the edge.

### 2.5. Calculation Principle of Edge Height

According to the principle of stereo vision [37], if two cameras acquire a pair of images of the same object at the same time, the spatial landscape of the object can be reproduced and its three-dimensional coordinates can be plotted as well [38]. Based on this principle, in photogrammetry, in order to obtain the position coordinates of a certain object, it is necessary to use two images with overlapping areas to form a stereopair [35].

**Figure 5:**
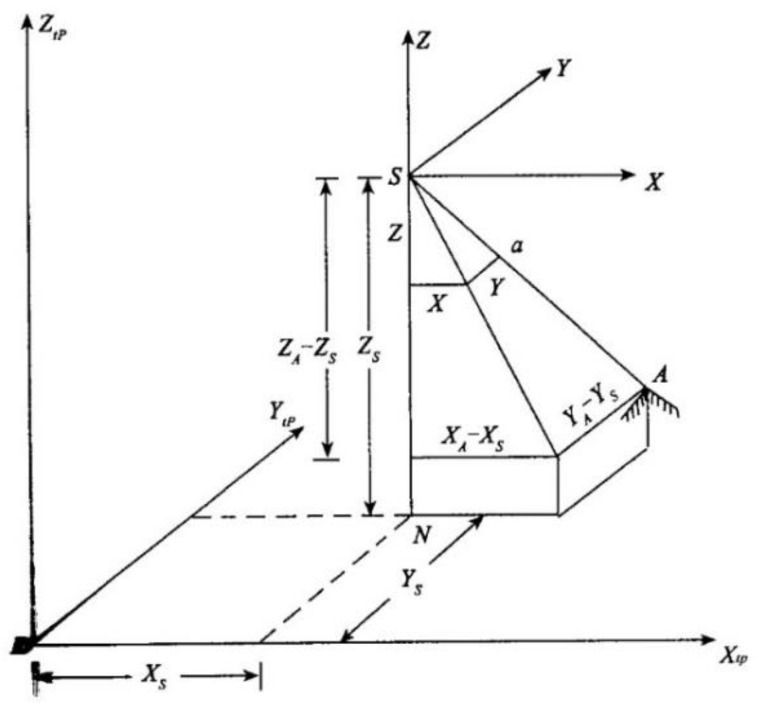
Schematic diagram of collinear equation.

As shown in Figure 5 [35], point S is the projective center of photography, and its coordinates in the object space coordinate system are $\left(X_{s}\;,Y_{s}\;,Z_{s}\right)$. Point A is a point of the rice on the platform, and its object space coordinates are $\left(X_{A}\;,Y_{A}\;,Z_{A}\right)$. Point a is the image of point A on the image, and its image space coordinates and image space auxiliary coordinates are (x , y , −f) and (X , Y , Z) respectively. According to the position relationship that point S, A and a are collinear in the moment of photography, the following relation exists:

(2) $$\frac{X}{X_{A}-X_{s}\;}=\frac{Y}{Y_{A}-Y_{s}}=\frac{Z}{Z_{A}-Z_{s}}=\lambda$$

λ is the scale factor.

There is the following relation between the image space auxiliary coordinate system and the image space coordinate system, that is, the image space coordinate system is obtained by rotating the image space auxiliary coordinate system around the Y, X and Z axis by φ, ω and κ three angles in turn. With Equation (2), we can obtain the following equation [39]:

(3) $$\begin{array}{cc} & \left[\begin{array}{c} x-x_{0}\\ y-y_{0}\\ -f \end{array}\right]=R\left[\begin{array}{c} X\\ Y\\ Z \end{array}\right]=\lambda R\left[\begin{array}{c} X_{A}-X_{s}\\ Y_{A}-Y_{s}\\ Z_{A}-Z_{s} \end{array}\right]=\lambda R_{\varphi }R_{\omega }R_{\kappa }\left[\begin{array}{c} X_{A}-X_{s}\\ Y_{A}-Y_{s}\\ Z_{A}-Z_{s} \end{array}\right]\\ = & \lambda \left[\begin{array}{ccc} \cos\varphi & 0 & -\sin\varphi \\ 0 & 1 & 0\\ \sin\varphi & 0 & \cos\varphi \end{array}\right]\left[\begin{array}{ccc} 1 & 0 & 0\\ 0 & \cos\omega & -\sin\omega \\ 0 & \sin\omega & \cos\omega \end{array}\right]\left[\begin{array}{ccc} \cos\kappa & -\sin\kappa & 0\\ \sin\kappa & \cos\kappa & 0\\ 0 & 0 & 1 \end{array}\right]\left[\begin{array}{c} X_{A}-X_{s}\\ Y_{A}-Y_{s}\\ Z_{A}-Z_{s} \end{array}\right]\\ = & \lambda \left[\begin{array}{ccc} \alpha_{1} & \beta_{1} & \gamma_{1}\\ \alpha_{2} & \beta_{2} & \gamma_{2}\\ \alpha_{3} & \beta_{3} & \gamma_{3} \end{array}\right]\left[\begin{array}{c} X_{A}-X_{s}\\ Y_{A}-Y_{s}\\ Z_{A}-Z_{s} \end{array}\right] \end{array}$$

Matrix R is the rotation matrix and related to the angle elements of exterior orientation, which are the three parameters determining the space attitude of the image in the moment of photography;

$\alpha_{i}$, $\beta_{i}$, $\gamma_{i}$(i=1,2,3) are the direction cosine composed of three angle elements of the elements of exterior orientation of the image;

$x_{0}$, $y_{0}$, f are the elements of interior orientation of the image (f is the principal distance of the camera, $x_{0}$ and $y_{0}$ are the coordinates of the principal point).

By expanding, dividing and rearranging of Equation (3), we have the following collinear equation:

(4) $$\{\begin{array}{c} x-x_{0}=-f\frac{\alpha_{1}\left(X_{A}-X_{s}\right)+\beta_{1}\left(Y_{A}-Y_{s}\right)+\gamma_{1}\left(Z_{A}-Z_{s}\right)}{\alpha_{3}\left(X_{A}-X_{s}\right)+\beta_{3}\left(Y_{A}-Y_{s}\right)+\gamma_{3}\left(Z_{A}-Z_{s}\right)}\\ y-y_{0}=-f\frac{\alpha_{2}\left(X_{A}-X_{s}\right)+\beta_{2}\left(Y_{A}-Y_{s}\right)+\gamma_{2}\left(Z_{A}-Z_{s}\right)}{\alpha_{3}\left(X_{A}-X_{s}\right)+\beta_{3}\left(Y_{A}-Y_{s}\right)+\gamma_{3}\left(Z_{A}-Z_{s}\right)} \end{array}$$

The collinear equation describes the relationship among the object space coordinates, image space coordinates and the elements of interior and exterior orientation.

**Figure 6:**
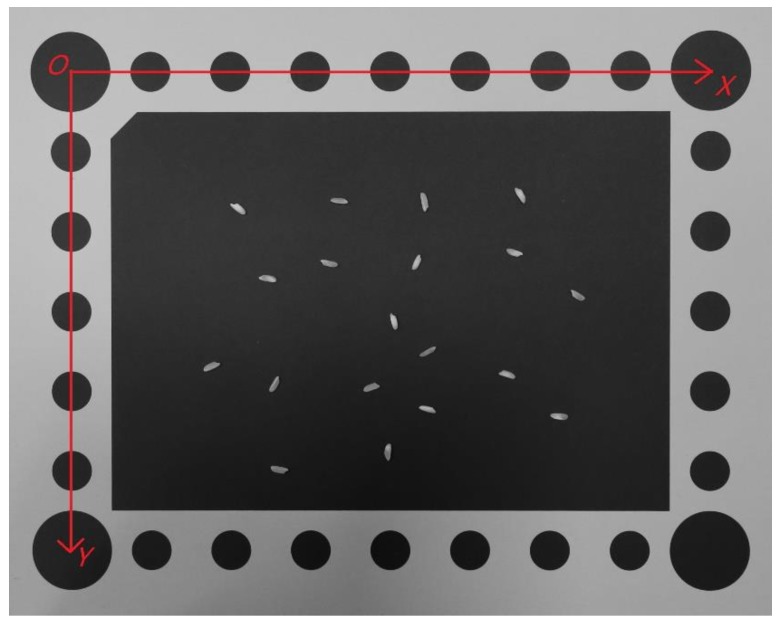
One of the images of rice.

One of the images obtained in the experiment is shown in Figure 6. In the object space, the object space coordinate system is constructed by taking the center O of the circle at the upper left corner of the background board as the origin, the plane on which the background board is located as the XOY plane, the long side of the background board as the X axis, the short side as the Y axis, and the axis perpendicular to the XOY plane through the origin as the Z axis. The radius of each circle and the distance between each center of the circle are known, so the object space coordinates of all the centers of circles are known as well. In the image, the row and column numbers of each center of the circle are extracted by the centroid detection algorithm as the image space coordinates of the center of the circle. Therefore, several control points with known object space coordinates and image space coordinates are obtained. According to Equation (4), the collinear equations listed by those control points can be used to calculate the elements of exterior orientation of the image. This process is called the single image space resection.

For a certain point on the rice, it generates two corresponding image points on the left and right images, which are called the corresponding points. The image space coordinates are determined by the row and column numbers of the corresponding points, and the elements of exterior orientation of the two cameras have been obtained before. So, based on Equation (4), using a pair of the corresponding points in the stereopair, four equations can be listed and three unknowns, which are the object space coordinates of the ground point corresponding to the pair of corresponding points, can be solved. This process is called stereopair space intersection.

According to the above analysis and the hypothesis of Section 2.4, the coordinates of rice edges such as the height of rice edges, can be calculated by matching the corresponding points of rice edges on the left and right images. The thickness of rice can be obtained indirectly by averaging the height of rice edges.

### 2.6. Matching the Corresponding Points on the Edge of Rice

**Figure 7:**
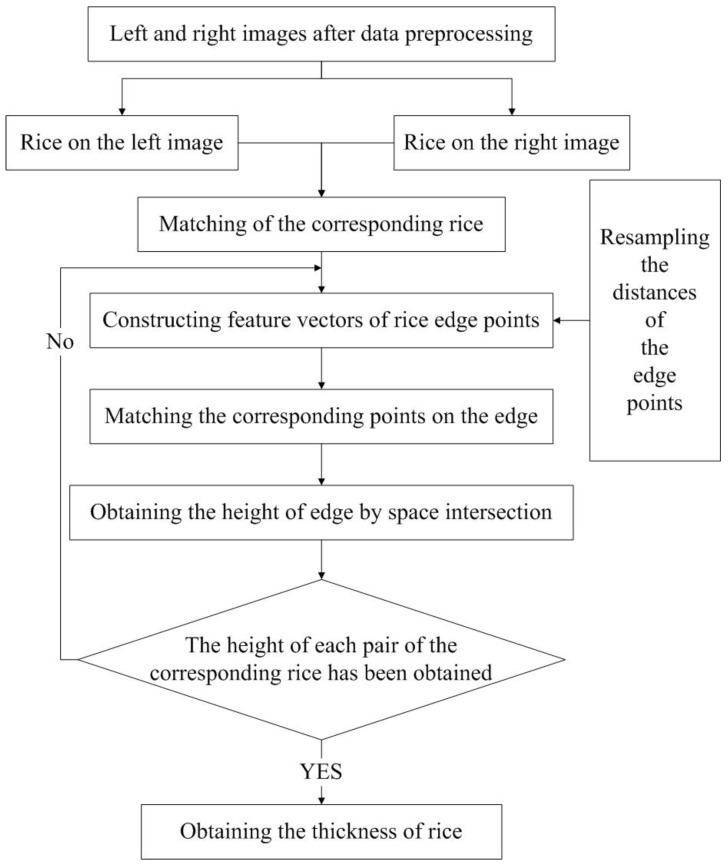
Flow chart for obtaining thickness.

The edge of rice is lack of texture features as well, which means it is difficult to directly match the corresponding points on the edges among several rice. The method adopted in this paper is to firstly match the corresponding rice, that is, to find the corresponding pair of rice on the left and right images, and secondly for each pair of corresponding rice, to construct feature vectors for each edge point. Finally, the corresponding points on the edges can be matched through the similarity of the feature vectors. The flow chart for obtaining the rice thickness based on the rice edge is shown in Figure 7.

#### 2.6.1. Matching the Corresponding Rice

**Figure 8:**
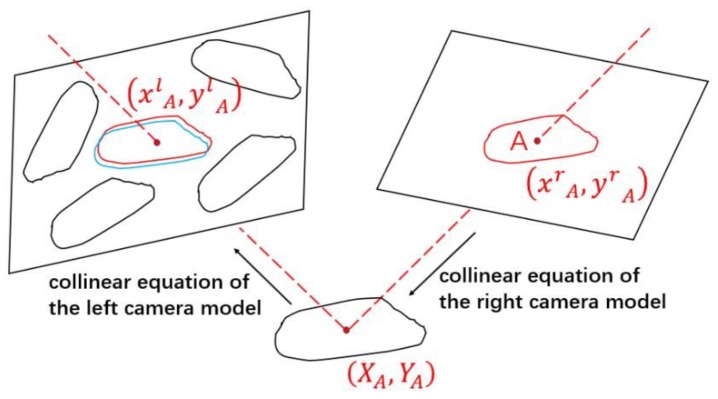
Process of matching the corresponding rice.

As shown in Figure 8, for the rice A on the right image (the red rice on the right image), the image space coordinates of its centroid are $\left(x^{r}{}_{A},\;y^{r}{}_{A}\right)$. The position of the rice $\left(X_{A},\;Y_{A}\right)$ are approximated by the collinear equation of the right camera model, which then we use as an approximation to guess the most probable correspondence as the corresponding rice in the left image (the blue rice on the left image) whose centroid falls closest to this point’s projection $\left(x^{l}{}_{A},\;y^{l}{}_{A}\right)$. In this way, the matching of all the corresponding rice on the left and right images will be completed.

#### 2.6.2. Constructing Feature Vectors of Edge Points and Matching the Corresponding Points

**Figure 9:**
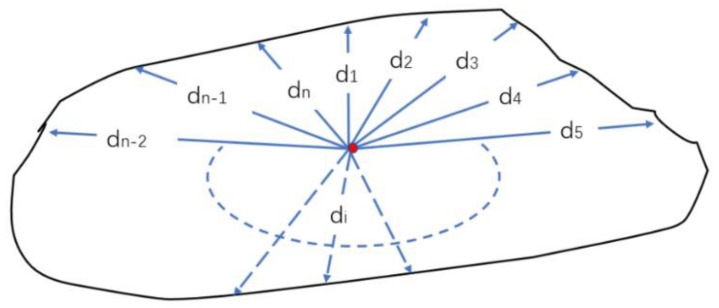
n distances from the centroid to edge points.

**Figure 10:**
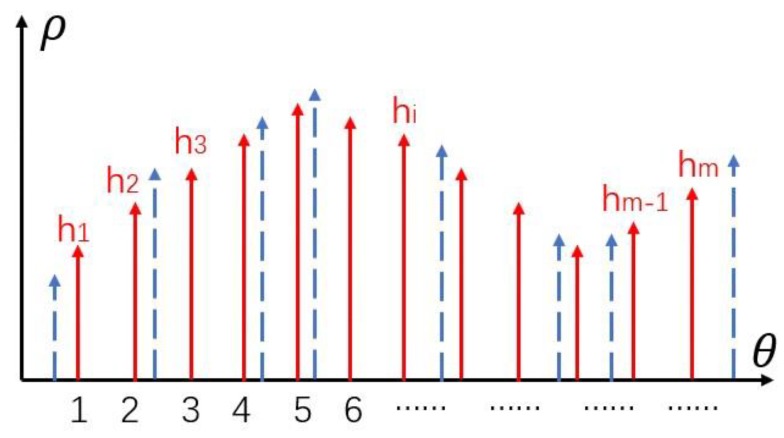
Resampling the distances.

For a pair of corresponding rice, we assume that there are n pixel points on one of the rice edges. Firstly, we carry on a resampling. The polar coordinate system is established with the centroid of rice as the origin, the half-line from the centroid to the right as the polar axis and counter clockwise as the positive direction. We define the intersection of the polar axis and the rice edge as the first point $p_{1}$, and then define the second point $p_{2}$, the third point $p_{3}$ until the n-th point $p_{n}$ in counter clockwise order. In Figure 9, n distances $d_{i}\;\left(i=1,2,3\cdots n\right)$ from the centroid to n edge points are calculated, respectively. Then the m distances $h_{i}\left(i=1,2,3\cdots m\right)$ are obtained by resampling the original n distances (blue dotted lines in the Figure 10) at intervals of 1 degree using linear interpolation.

Since there are m points on the left and right edge of rice respectively after resampling ($pl_{1},pl_{2},pl_{3},\ldots \ldots pl_{m}$ on the left edge and $pr_{1},pr_{2},pr_{3},\ldots \ldots pr_{m}$ on the right edge), the feature of the first edge point is defined as the vector $\left[h_{1},\;h_{2},\;h_{3},\;\cdots ,h_{m-1\;,}h_{m}\right]$; the feature of the second edge point is defined as the vector $\left[h_{2},\;h_{3},\;\cdots ,h_{m-1\;,}h_{m},\;h_{1}\right]$, likewise, the feature of the i-th (i=1,2,3⋯m) edge point is defined as the vector $\left[h_{i},\;h_{i+1},\;\cdots ,h_{m},\;h_{1},\;\cdots ,\;h_{i-1}\right]$.

Then the matching process of the feature vectors is as follows: given the feature vector $\left[h_{1},\;h_{2},h_{3},\cdots ,h_{m-1,}h_{m}\right]$ of the first point $pl_{1}$ on the left edge, the similarity is calculated with m feature vectors of the points on the right edge of the rice respectively. The similarity measure we used is the correlation coefficient method, so m correlation coefficients can be obtained. Suppose the correlation coefficient between $pl_{1}$ and $pr_{k}$ is highest, we decide that $pl_{1}$ and $pr_{k}$ is a pair of the corresponding points, $pl_{2}$ and $pr_{k+1}$ is another pair of the corresponding points, and so on. Therefore, we finished the matching of all edge corresponding points in an angular ordering.

There are two reasons why the resampling has to be done: (i) due to the influence of various shooting angles and other factors, the shapes of the same rice on the left and right images are slightly different. Therefore, the number of pixels on the left and right edges is inconsistent, which means that the lengths of the feature vectors differ from each other and the matching can’t be carried on. This problem can be solved by resampling to keep the number of points on the left and right edges consistent; (ii) resampling can realize the matching of subpixels, which means we can obtain more corresponding points on the edge than the original pixels so that the accuracy of the calculated height can be improved [40,41].

In addition, the reason why we choose 1 degree as the resampling interval is as follow: The smaller the resampling interval is, the more subpixel points can be obtained and the higher the accuracy of the calculated height. Suppose that the semi-major axis of the rice is about 5mm, the semi-minor axis is about 1mm, and the image resolution is about 0.1mm, then the arc length represented by the interval of 1 degree is between 0.27 and 0.87 pixels, which meets the requirements of subpixel sampling, that is, the number of edge points after resampling is more than the original number. If we continue to decrease the interval, we will obtain more subpixel points, which, however, will greatly increase the dimension of feature vectors and the calculation of the model. Therefore, we choose 1 degree as the resampling interval, which is a common and effective parameter number.

### 2.7. Obtaining the Height of the Edge by Space Intersection

After obtaining the corresponding points of the rice edges on the left and right images, the height of the rice edge points is calculated by using the space intersection described in Section 2.5. The average height of all the edge points is taken as the height of the rice edge. The thickness of rice is twice the average height of the rice edges.

## 3. Results and Discussion

### 3.1. Data Preprocessing

#### 3.1.1. Image Undistortion

For the projection, the farther the distance from the center of the lens is, the bigger the distortion of the image occurs. If the dimensional information is directly measured from the original image, there will be a large error. In order to eliminate the optical distortions introduced by the lenses, it is necessary to use the camera’s distortion coefficients to correct the distortion of the left and right original images.

#### 3.1.2. Rice Extraction from the Image

**Figure 11:**
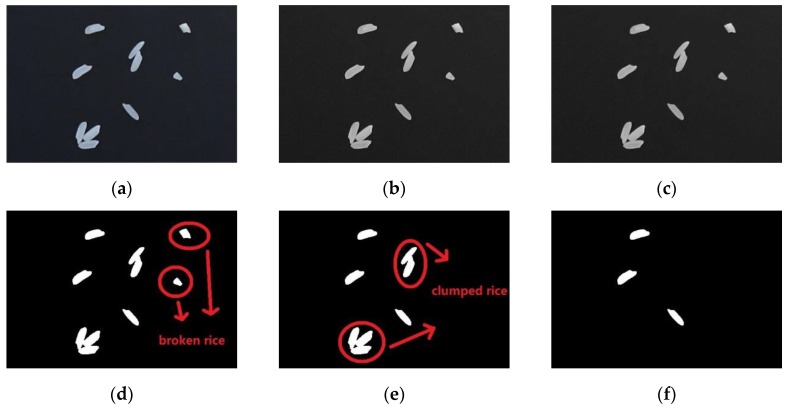
Steps of rice extraction from images: (**a**) original image; (**b**) image after graying; (**c**)image after filtering and denoising; (**d**) image after binarization; (**e**) removing broken rice; (**f**)removing clumped rice.

In order to avoid the interference of the background on the measurement of rice thickness, the rice in the image were extracted, which means the gray level of the non-rice part of the image was assigned to 0 as the background. The specific steps are as follows:

(1) Image graying. Original colorful images (Figure 11a) were stored through red-blue-green (RBG) channels, however the gray level information was not needed. The image was grayed firstly, which converts RGB values to grayscale values by forming a weighted sum of the R, G, and B components: 0.2989 × R + 0.5870 × G + 0.1140 × B (Figure 11b).
(2) Filtering and denoising. In the process of image acquisition, due to the interference of camera itself or external illumination, dust on the background platform and so on, there is noise in the image. The median filtering method was used to de-noise (Figure 11c).
(3) Binarization. Otsu’s algorithm [42] was adopted to binarize the image. Pixel value of background was 0 and the target (rice) was 1 (Figure 11d).
(4) Image postprocessing. In fact, there may be broken rice kernels in the rice samples, or two or even more rice may clump together and stick together [43]. By judging the area of each object, the threshold was set to remove the objects with too small or too large area directly, so as to ensure that the object studied were not disturbed by accidental error samples (Figure 11e,f).

### 3.2. Visualization of the Matching of Corresponding Points

**Figure 12:**
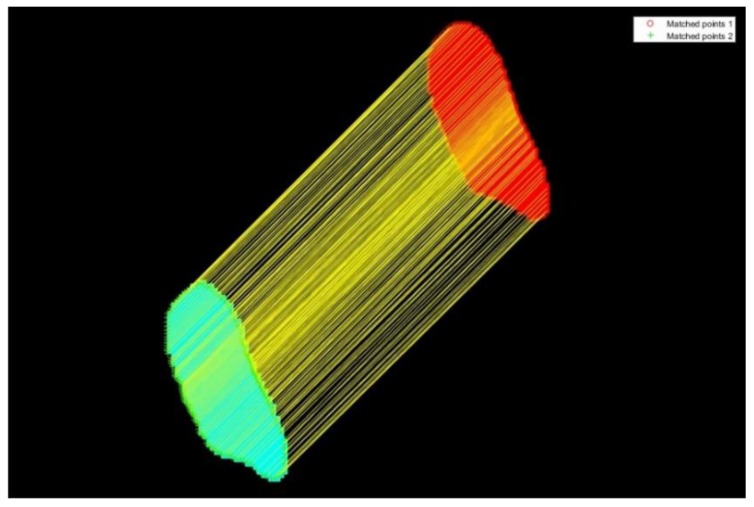
Matching of the corresponding points.

After data preprocessing, the algorithm automatically matched the corresponding rice firstly, and then the corresponding points on the rice edges were matched between each pair of corresponding rice. Figure 12 shows the visualized matching of corresponding points on the edges of one pair of corresponding rice. Each pair of the corresponding points is connected by a yellow line. It can be judged by visual interpretation that the matching of the corresponding points is correct, which proves this matching algorithm of constructing features based on distance vectors is feasible.

### 3.3. Accuracy of Thickness Extraction

The height of each rice edge was calculated and the thickness of rice was further obtained. Table 2 shows the comparison between the calculated thickness value of the algorithm and the true value of manual measurement. Experiments were carried out on six kinds of rice or grain. For each type of rice, the operation was repeated three times.

As can be seen from the table, the time spent in each sample averagely was about 1.05 s, and the time spent in calculating the thickness of one kind of rice was almost around 21 s, which was much faster than that of manual measurement.

**Figure 13:**
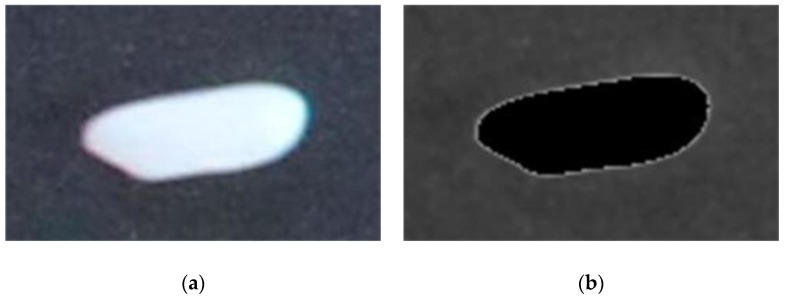
Result of rice extraction: (**a**) original rice image; (**b**) the extracted part (rice) was filled with black, but still a small part of white remained.

The error between the calculated value and the measured value was also very small. The maximum error and the minimum error of thickness were 0.10 mm and 0.01 mm, respectively. According to the national industry standard LS-T 6116-2017 [44], the error of no more than 0.1 mm was satisfied, which proved the accuracy of the algorithm for extracting rice thickness. In addition, most of the calculated values were greater than the measured values. According to the result of rice extraction (Figure 13), the part of the original image was filled with black if it was extracted as rice, but it can be seen that the edge of rice extracted was not exactly the actual edge of rice, but the one slightly narrowed at the center. Since the height of each point on the rice surface increases from the edge to interior, the height we obtained was greater than the actual height, which may be the reason why the calculated values were greater than the measured values.

For the rice grain, the errors of thickness were only 0.08, 0.08 and 0.04 mm, which showed that the algorithm was also applicable to the rice grain.

### 3.4. Selection of the Amount of Samples

The average thickness of manual measurement was used as the true value of thickness. In terms of the calculated result of image processing, theoretically, selecting a large enough or even infinite number of samples, the average calculated value, which is the average value of 20 calculated thickness in this paper which we called the ‘average of the large sample set’ will be close enough to the ideal true value. However, given the algorithm time and practical operation difficulty, it is impossible to use a too large nor an infinite number of samples in an experiment. We hope to find an appropriate amount which is as small as possible, and the thickness extracted with this amount of samples can be close to the ‘average of the large sample set’.

**Figure 14:**
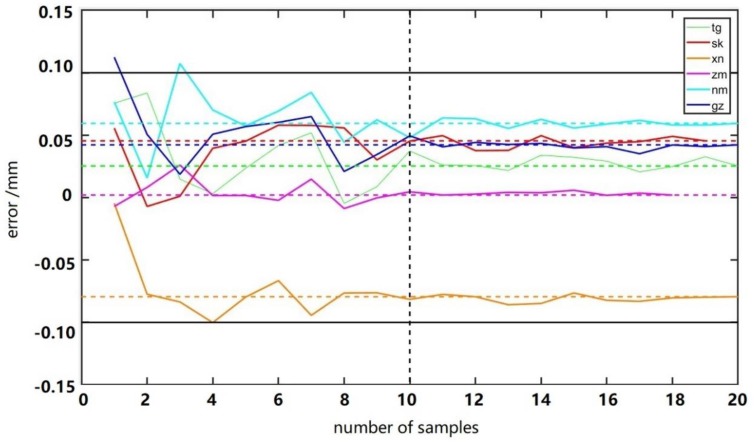
The approximation degree between the true value and the calculated value extracted from different amounts of samples.

The average value of 20 manual measured values was taken as the true value of their respective thickness. Figure 14 shows the approximation degree between the true value and calculated value extracted from different amounts of rice or grains. The abscissa represented the number of samples randomly selected, and the ordinate represented the error between the calculated thickness and the true value with the corresponding amount of samples. In order to ensure that the error was not accidental, each error value was the average value of repeated experiments with the corresponding number of samples. The black horizontal line represented the upper limit of error. The error not exceeding this range was acceptable. The horizontal dashed line represented the error between the ‘average of the large sample set’ and the true value. We called it ‘error of the large sample set’. Firstly, it can be seen that the thickness errors were almost less than 0.1 with any sample amount, which indicated that the thickness extracted with these amounts was reliable, and proved the reliability of the thickness algorithm again. Secondly, as the number of samples increased, the error fluctuated around the respective dashed lines and became increasingly closer. By comparison and observation, the thickness errors of all kinds of rice and grain were all almost close enough to the ‘error of the large sample set’ when about 10 or more rice was taken as the sample amount, indicating that when the thickness was extracted with 10 rice samples, although the accuracy was slightly lower than that of more samples, it had met the accuracy requirements and the operation was much more efficient. In the national industry standard, it is suggested that 10 should be taken as the sample amount in the extraction of rice dimensions parameters by way of image processing [44], with which the conclusion of this paper was consistent.

### 3.5. Effect of the Base–Height Ratio

**Figure 15:**
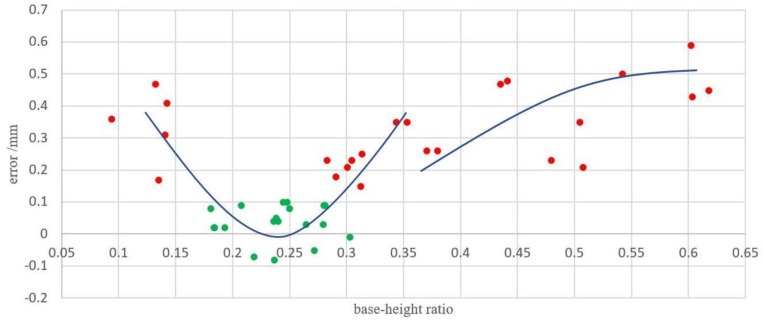
Effect of the base-height ratio on thickness accuracy.

In this study, 43 base–height ratios were selected to test the effect on the thickness accuracy (we only change the baseline while the distance from the ground plane to the cameras remains unchanged during the experiment, at which height, it is ensured that all rice can be photographed with the highest image resolution). Figure 15 shows the errors between the calculated values and measured values with different baselines ranging from about 25 to 250 mm. With the increase in base–height ratio, the error first decreased rapidly and then increased sharply, and tended to do so gently when the base–height ratio became bigger and bigger. Since the upper limit was 0.1mm, the accuracy was accepted (green dots in Figure 15) when the base–height ratio was about 0.18 to 0.28, which belongs to small base-height ratio. Small base–height ratio images are effective when the surface is wavy obviously, which is consistent with our platform covered with rice.

## 4. Conclusions

An automatic extraction method of rice thickness based on edge features was proposed in this paper. The hypothesis that the average height of the edges of multiple rice is half of the thickness of rice was put forward, which transformed the target from the thickness of rice to the height of the edge, so the problem that the corresponding points of the highest points of rice cannot be directly matched due to the scare texture of rice surface was solved. The method used the shape of the edge instead of the texture of the edge as features to match the corresponding edge points, which solved the problem caused by the structural similarity of edge. Compared with the measured true values, the maximum and minimum errors of thickness are 0.10 and 0.01 mm, which meets the requirements, indicating the reliability of the method. The appropriate amount of samples and base-height ratio were also important factors which may affect the accuracy.

This paper still has some further research directions. First, since the edge features are the decisive factor of the method which may easily be affected by many conditions such as illumination, a further study on edge extraction is needed, which can improve the accuracy of the method. Second, since we have a stereo configuration, we can use epipolar geometry to match the corresponding points on the rice edge, which can simplify our matching procedure and speed up the process. We do not do it in this paper because using our matching as it is now proves that the feature vector computed is discriminative enough, and when we compute the minimum correlation between $pl_{1}$ and all the candidate $pr_{k}$, the true matching can be found. We will using epipolar geometry in our future research.

## Figures and Tables

**Table 1:** Introduction of experimental samples.

No.	Name (Abbr.)	Category	Source	Appearance Characteristics
1	Thai rice (tg)	Indica rice	market	slender rice shape; high transparency
2	Shengke rice (sk)	Indica rice	Hainanexperiment field	long shape
3	Xianning rice (xn)	Japonica rice	Xianning experiment field	medium rice shape
4	Zaomi rice (zm)	Japonica rice	Xianning experiment field	short and full
5	Glutinous rice (nm)	Sticky rice	market	medium rice shape; low transparency
6	Luoyou9348 (gz)	Indica rice grain	Ezhou experiment field	slender grain shape with two pointed ends

**Table 2:** Errors between calculated values and measured values.

Rice/Grain	No.	Rice Thickness			Time Consuming (s)	Time Consumed by Each Sample (s)
		Calculated Value/mm	Measured Value/mm	Error/mm		
tg	1	1.73	1.63	0.10	20.96	1.05
	2	1.65		0.02	20.77	1.04
	3	1.65		0.02	21.19	1.06
sk	1	1.76	1.83	−0.07	21.07	1.05
	2	1.86		0.03	21.01	1.05
	3	1.93		0.10	21.29	1.06
xn	1	1.85	1.77	0.08	20.86	1.04
	2	1.81		0.04	20.91	1.05
	3	1.86		0.09	20.90	1.05
zm	1	1.91	1.86	0.05	21.21	1.06
	2	1.85		−0.01	21.41	1.07
	3	1.87		0.01	20.96	1.05
nm	1	1.87	1.82	0.05	20.86	1.04
	2	1.78		−0.04	21.01	1.05
	3	1.88		0.06	20.89	1.04
gz	1	2.23	2.15	0.08	21.20	1.06
	2	2.23	2.15	0.08	20.95	1.05
	3	2.13	2.09	0.04	20.91	1.05
